# Evaluation of the therapeutic effects of smoking cessation on chronic central serous chorioretinopathy

**DOI:** 10.1038/s41598-025-10628-4

**Published:** 2025-07-24

**Authors:** Tatsuo Nagata, Nobuhisa Ochiai, Hiroyuki Kondo, Kazuma Oku, Mizuki Tsurusaki, Takuma Futami, Akihisa Watanabe, Itsuka Matsushita

**Affiliations:** Department of Ophthalmology, University of Occupational and Environmental Health, Japan, Kitakyushu, Japan

**Keywords:** Retinal diseases, Risk factors, Medical research

## Abstract

Although there have been reports that cigarette smoking is a risk factor for the development of central serous chorioretinopathy (CSCR), there have been no reports that have determined whether smoking cessation is helpful in the resolution of CSCR. The purpose of this study was to evaluate the effects of smoking cessation on chronic CSCR. Fifty-two eyes of 45 patients (22 smokers and 23 nonsmokers) diagnosed with chronic CSCR at our hospital from January 2016 to February 2023 were studied. All patients with CSCR were instructed to take lutein supplements and, if they were smokers, to quit. Among the 52 eyes with chronic CSCR, 33 (63.5%) improved after that instruction, 14 (26.9%) required a laser for CSCR resolution, and 5 (9.6%) had persistent CSCR. Among the 22 smokers who received smoking cessation instructions, 16 stopped smoking, and 6 reduced their smoking to one or two per day. The rate of improvement without laser photocoagulation in chronic CSCR was significantly higher in smokers (77.8%) than in nonsmokers (48.0%) (*p* = 0.026). The results indicate that chronic CSCR patients who smoke have a higher rate of improvement when they quit smoking than CSCR patients who do not smoke. This study is the first to demonstrate that reducing the risk factor for smoking is a useful initial treatment for CSCR patients who smoke.

**Supplementary Information:**

The online version contains supplementary material available at 10.1038/s41598-025-10628-4.

## Introduction

Central serous chorioretinopathy (CSCR) is characterized by serous retinal detachment (SRD) with dysfunction of the choroid and retinal pigment epithelium. The CSCR can be divided into acute and chronic stages, but the differences between them have not been clearly determined. Generally, acute CSCR is characterized by SRD that resolves within 3 months, whereas chronic CSCR is characterized by SRD that lasts more than 4–6 months^1^. Since there have been no gold standard diagnostic criteria for CSCR, in 2020 Chhablani et al. proposed criteria based on multimodal imaging. Among them, bullous variant, RPE tear, and those associated with other ophthalmic diseases are classified as Atypical and separate from typical CSCR^2^. Acute CSCR usually progresses to spontaneous remission after 2–3 months of follow-up, but in long-term chronic cases, after the retinal pigment epithelial barrier lesion is identified by retinal angiography, not only direct photocoagulation but also MicroPulse^®^ laser, selective retina therapy (SRT), photodynamic therapy (PDT) and reduced-fluence PDT(RF-PDT) have recently been used. In Japan, PDT is not covered by public insurance and is not approved by the government, so treatment may be difficult if the leakage point is in the macular area or if there is no obvious leakage point on retinal angiography. As for conservative therapy, there are reports that oral acetazolamide^3^ and lutein supplements^4^ have been effective in reducing SRD in chronic CSCR.

Structural abnormalities of the choroidal vessels and vascular autoregulation may be responsible for the development of CSCR^5^. Then, a substantial proportion of acute CSCR cases may undergo spontaneous remission. However, the underlying pathogenesis that determines which cases progress to the chronic phase remains uncertain. Although it is known that there is a causal relationship between the development of a CSCR and cigarette smoking^6,7^, no study has examined the effect of smoking cessation on CSCR. In other words, if smoking contributes to the development of CSCR, it remains uncertain whether smoking cessation can reverse serous retinal fluid (SRF) accumulation. Thus, the purpose of this study was to evaluate the effects of smoking cessation on eyes with chronic CSCR.

## Methods

The Ethics Review Committee of the University of Occupational and Environmental Health (UOEH), Kitakyushu, Japan, approved the study protocol (UOEHCRB23-026), and waived the need of obtaining informed consent due to the retrospective nature of the study. The study was conducted in accordance with the tenets of the Declaration of Helsinki. This was an observational study of 52 eyes of 45 patients diagnosed with chronic CSCR with SRD lasting more than 4 months from onset. Typical CSCR cases were selected, and patients whose cause was atypical CSCR, age-related macular degeneration, macular pit syndrome, diabetes, diseases requiring corticosteroids, or possible renal dysfunction were excluded from the study. All patients were examined at UOEH Hospital from January 2016 to February 2023; all were naïve cases and had not received any treatment or guidance before they were first examined at our hospital. Fifty-two eyes of 45 CSCR patients were divided into 27 eyes of 22 patients who were current smokers and 25 eyes of 23 patients who were current nonsmokers. The status of smoking was defined as “current smoker”, an individual who was smoking cigarettes at that time; “never smoker”, an individual who had never smoked; and “former smoker”, an individual who had already quit smoking prior to the onset of CSCR. The Brinkman index (BI), which is equal to (number of cigarettes per day) × (number of years of smoking), was used to calculate lifetime exposure to smoking^8^.

At the initial visit to our institution, all CSCR patients were instructed to supplement lutein at least 10 mg/day, and if they were smokers, they were also instructed to quit smoking and were followed up as conservative treatment. The time of onset of CSCR was based on the findings of the referring ophthalmologist and an interview with each patient.

The patient and family members were interviewed about their smoking cessation status and supplement inoculation status, and if the patient had difficulty quitting smoking on their own, they were referred to a smoking cessation outpatient clinic. Comprehensive ocular examinations, including measurements of best-corrected visual acuity (BCVA) via a Landolt ring chart and optical coherence tomography (OCT; DRI OCT Triton, Topcon, Tokyo, Japan), were performed at each visit. Fundus fluorescein angiography (FA) and fundus indocyanine angiography (IA) with a Heidelberg Retina Angiograph (Heidelberg Spectralis; Heidelberg Engineering, Heidelberg, Germany) were performed at the initial visit or when laser photocoagulation therapy (PC) was needed.

The following data were analyzed: smoking habit, Brinkman index (BI), smoking cessation, direct PC, interval from onset to first visit to our hospital, interval from onset to SRD disappearance, refractive error (spherical equivalent), BCVA measurements, height of the SRD, and central choroidal thickness (CCT) at the initial and final examinations. The heights of the foveal retinal detachment and choroidal thickness in the OCT images were measured manually via the caliper function of the OCT device.

Direct PC was performed in chronic CSCR patients whose SRD had not decreased 3 months after their visit to our institution and whose leak point was confirmed by FA. The percentage of eyes whose SRD resolved without laser treatment was statistically examined. Patients whose SRD did not disappear at least 12 months after the first visit to our hospital were classified as having persistent CSCR.

### Statistical analyses

All the statistical analyses were performed via JMP version 11.2.0 software (SAS Inc., Cary, NA, USA). The data were analyzed via descriptive statistics. The BCVA measured in decimal units was converted to the logarithm of the minimal angle of resolution (logMAR) for the statistical analyses. Age, Duration from onset to first visit our institution, BCVA, Spherical Equivalent Value, Initial SRD height, Initial CCT, were compared between smokers and nonsmokers (Mann-Whitney U test). Gender at first visit to our institution was also compared between smokers and nonsmokers (Fisher’s exact test). The mean BCVA at the final visit was compared to the baseline BCVA at the initial examination (Wilcoxon rank sum test). The rate of resolution of SRD without Direct PC in chronic CSCR patients were compared between the smoker and nonsmoker groups (Pearson’s chi-square test). The mean CCT of all patients was compared before treatment and at the final examination (Wilcoxon rank sum test). The changes in the mean CCT and the mean SRD for smokers and nonsmokers were subsequently compared (Wilcoxon rank sum test). A multivariate analysis of the measured data was performed via logistic regression analysis to determine the factors associated with improved SRD in CSCR patients. The odds ratio (OR), confidence interval (CI) and *p* value were calculated. Statistical significance was set at *p* < 0.05.

## Results

Fifty-two eyes of 45 CSCR patients, consisting of 42 eyes of 36 males and 10 eyes of 9 females, were studied. The mean age was 48.6 ± 10.3 years. The demographic and clinical characteristics of the study population are shown in Table 1. The mean refractive error (spherical equivalent) of all the CSCR patients was − 0.58 ± 1.54 diopters (D). Table 2 shows a comparison of the baseline characteristics of current smokers and current nonsmokers. There were no significant differences in Age, Duration from onset to first visit our institution, initial BCVA, spherical equivalent value, initial SRD height, and initial CCT between current smokers and current nonsmokers (Mann-Whitney U test, respectively). However, the proportion of males who were current smokers was significantly higher (*p* < 0.001, Fisher’s exact test).

**Table 1:** Demographic and clinical characteristics of the study Population.

Characteristics	Values
Mean age, years	48.6 ± 10.3
Male (%)	36(80.0)
Current smokers (%, gender)	22(48.9, all male)
Currently nonsmokers (%, gender)	23 (51.1, 12 male and 11 female)
Former smokers (%, gender)	7 (15.6, 6 male and 1 female)
Never smoked before (%, gender)	16 (35.6, 6 male and 10 female)
Duration from onset to first visit our institution, months	4.77 ± 5.47
Duration of existence of SRDs, months (The exception of 5 persistent CSCR eyes)	7.85 ± 3.75

**Table 2:** Comparison of baseline characteristics between current smokers and current Nonsmokers.

Characteristics	Current smoker	Currently nonsmoker	*p* value
Age, years	48.0 ± 9.0	49.1 ± 11.6	0.570 Mann‒Whitney U test
Gender, Male (%)	22(100)	12(52.2)	< 0.001 Fisher’s exact test
Duration from onset to first visit our institution, month	3.37 ± 2.68	6.28 ± 7.16	0.740 Mann‒Whitney U test
Initial BCVA, logMAR	0.12 ± 0.21	0.144 ± 0.27	0.856 Mann‒Whitney U test
Spherical Equivalent Value, Diopter	−0.45 ± 1.20	−0.71 ± 1.84	0.780 Mann‒Whitney U test
Initial SRD height, µm	138.4 ± 123.6	143.2 ± 98.3	0.634 Mann‒Whitney U test
Initial CCT, µm	420.1 ± 142.6	368.0 ± 120.8	0.203 Mann‒Whitney U test

**Fig. 1:**
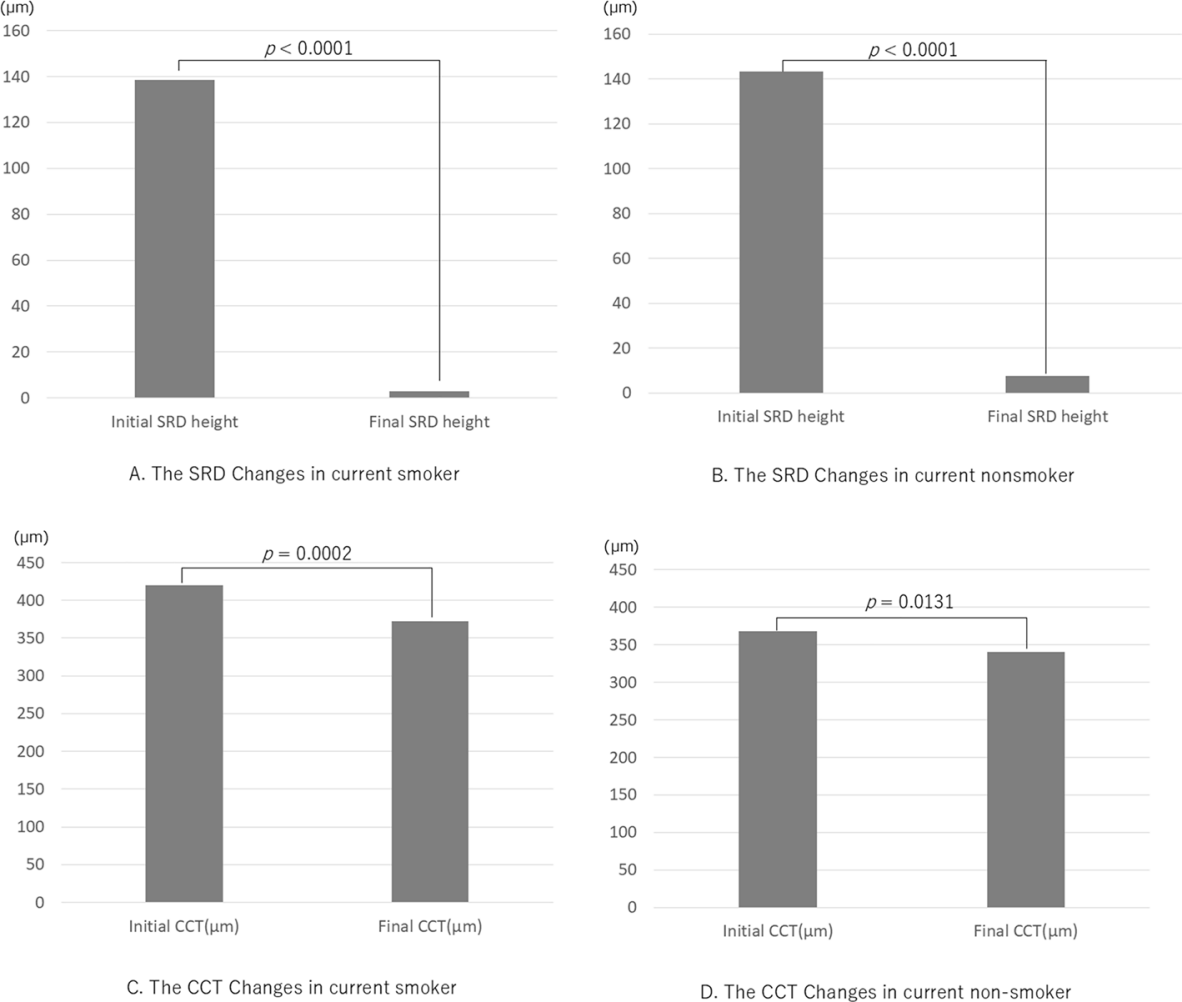
Changes in serous retinal detachment (SRD) height and central choroidal thickness (CCT) in smokers and nonsmokers with chronic CSCR. Compared with the initial visit and the final visit, the mean SRD for both current smokers **(A)** and nonsmokers **(B)** was significantly lower, and the mean CCT for both current smokers **(C)** and nonsmokers **(D)** was also significantly lower (Wilcoxon rank sum test for all).

The smoking history, course of disease findings, and visual function of all patients are summarized in the supplementary data (Table S1). The mean BCVA improved significantly from 0.13 ± 0.24 logMAR units at baseline to 0.00 ± 0.25 logMAR units at the final visit (*p* < 0.001, Wilcoxon rank sum test). Twenty-two patients (48.9%, all male) were current smokers at the time of the initial examination and had a mean BI of 525.5. Seven patients (15.6%, 6 males and 1 female) were former smokers, all of whom had quit smoking more than five years earlier, with a mean Brinkmann index of 402.9. The remaining 16 patients (35.6%, 6 male and 10 female) had never smoked. The mean age of the current smokers was 48.0 ± 9.0 years, and that of the current nonsmokers (former + never) was 49.1 ± 11.6 years, with no significant differences (*p* = 0.570, Mann‒Whitney U test). Of the 22 current smokers, 16 stopped smoking cigarettes completely, and 6 reduced their number of cigarettes smoked from 10 or more per day to 1–2 per day after the smoking cessation instructions were provided. Due in part to the explanation of the risks of smoking and the possible benefits of lutein supplements to the patient and his/her family, and in part to the collaboration with the smoking cessation physician, there were no dropouts among the chronic CSCR patients picked up in the study. The mean CCT of all patients was 395.1 ± 133.9 μm at the first visit and 357.1 ± 129.4 μm at the final examination. The decrease was significant (*p* < 0.001, Wilcoxon rank sum test). The mean SRD of current smokers decreased significantly from 138.4 ± 123.6 μm to 2.81 ± 14.6 μm, and that of nonsmokers decreased from 143.2 ± 98.3 μm to 7.48 ± 20.7 μm (*p* < 0.001 for each, Wilcoxon rank sum test; Fig. 1A, B). The mean CCT of current smokers decreased significantly from 420.1 ± 142.6 μm to 372.6 ± 141.2 μm, and that of nonsmokers decreased from 368.0 ± 120.8 μm to 340.3 ± 115.8 μm (*p* = 0.0002 and *p* = 0.0131, respectively, Wilcoxon rank sum test, Fig. 1C, D). The CCT increased in 8 eyes (14.8%), including 3 PC-treated eyes and 2 persistent CSCR eyes.

Analysis of 52 eyes with chronic CSCR revealed that SRD resolved with conservative treatment in 33 eyes (63.5%), 14 eyes (26.9%) required direct PC to resolve SRD, and 5 eyes (9.6%) with persistent CSCR had residual SRD. Logistic regression analysis of variables affecting the resolution of SRD revealed a younger age (OR: 1.10; 95% CI: 1.01–1.19; *p* = 0.035), shorter time between onset and first visit our institution (OR: 1.28; 95% CI: 1.06–1.54; *p* = 0.01), and lower final height of the CCT (OR: 1.01; 95% CI: 1.00–1.01; *p* = 0.022). The five eyes (9.6%) with residual SRD were persistent CSCRs whose SRD had not resolved after more than one year from the initial diagnosis at our hospital. None of the 5 eyes had a clear leakage site on retinal angiography, so PC could not be performed. The SRD height of the patients with persistent CSCR did not change in three eyes and decreased in two eyes.

**Fig. 2:**
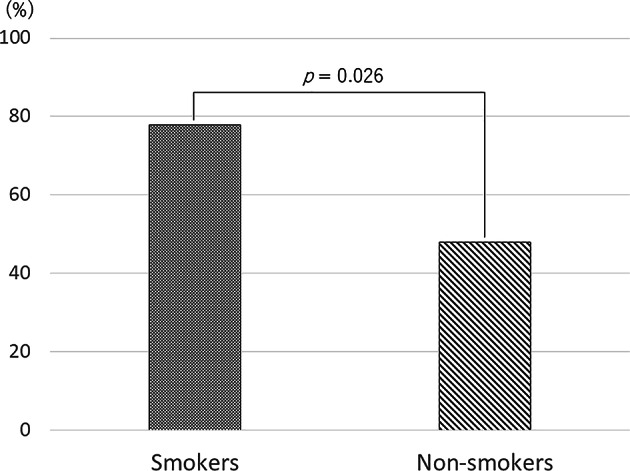
Differences in the rate of resolution of SRD without laser treatment between smokers and nonsmokers with chronic CSCR. Among the 27 eyes of smokers with chronic CSCR, the SRD disappeared in 21 eyes after smoking cessation and lutein supplementation (77.8%). Among the 25 eyes of nonsmokers with chronic CSCR, the SRD disappeared in 12 eyes after lutein supplementation (48.0%). This difference was significant (*p* = 0.026, Pearson’s chi-square test).

Among the 27 eyes of 22 current smokers, 21 eyes (77.8%) experienced SRD resolution after smoking cessation and lutein supplementation. Five eyes (18.5%) had SRD that resolved after direct PC, and 1 persistent CSCR eye (3.7%) with residual SRD was untreatable with direct PC for no clear leakage point on retinal angiography. The SRD disappearance rate without direct PC in the current smoker group was significantly greater than that in the current nonsmoker group (77.8% and 48.0%, respectively, *p* = 0.026, Pearson’s chi-square test, Fig. 2).

## Discussion

Smoking, steroid use, the use of antidepressants/anxiolytics, pregnancy, and hyperopia are known risk factors for CSCR^6,9^. Other risk factors associated with CSCR include gastroesophageal disorders such as *Helicobacter pylori* infections, uncontrolled systemic hypertension, the use of antibiotics, allergic respiratory diseases, high socioeconomic status, alcohol consumption, smoking, coronary heart disease, obstructive sleep apnea, poor sleep quality and autoimmune disease^10–18^. Earlier studies reported that myopia reduces the risk of CSCR^6^ but 59.6% of the eyes in our study had mild myopia (from − 0.5 D to −3.5 D). Clinically, CSCR is often difficult to treat because it can be caused by factors that are difficult to assess, such as psychological stress, or by multiple factors. The etiology of the five eyes with persistent CSCR with residual SRD in this study is also unknown. In those five eyes, more than one year had passed since the onset of CSCR at the time of our initial examination, and retinal thickness had already decreased. In addition, the leakage point could not be identified by FA or IA in all five eyes, so direct PC could not be performed. In Japan, PDT is not approved by the government and cannot be used to treat CSCR, but even if PDT or RF-PDT could be administered to the patients and was successful in eliminating their SRD, the sensory retina was already thinning, so it is likely that these patients would have had difficulty improvement in visual acuity. The cause of persistent CSCR may involve disruption of retinal function to transport water and vitreomacular traction. According to the logistic regression analysis of this study, younger age, a shorter time between onset and first visit our institution, and a lower final height of the CCT were factors associated with CSCR recovery. These findings indicate that it is important to receive a diagnosis and initiate treatment as early as possible to avoid chronic CSCR. Statistically, there was no significant difference in time to first visit to our institution between smokers and nonsmokers, but there were more nonsmokers in the persistent CSCR cases (4/5 cases). The small number of cases makes it difficult to analyze the results, but it is possible that this is because nonsmokers are more likely to have persistent CSCR, or that the cases that took more than a year from onset to our hospital visit happened to be more in the nonsmoker group.

Lutein is a member of the xanthophyll carotenoid family and is found mainly in the inner plexiform layer and in Henle’s fiber layer^19^but it can also be found in Müller cells. Lutein is thought to protect the eye by absorbing blue light and removing reactive oxygen species because of its high antioxidant activity^19,20^. It has been suggested that the antioxidant defense system may be impaired in eyes with CSCR^21^. Patients with CSCR have been reported to have lower plasma lutein concentrations than healthy subjects^22^. Therefore, lutein supplementation and smoking cessation may protect the RPE from oxidative stress and improve the CSCR. Oral lutein supplementation has been reported to be helpful in improving CSCRs^4^. From these results, we recommend at least 10 mg/day of lutein supplementation for not only chronic cases but also all CSCR cases at our institution. While it is difficult to compare acute CSCR because it often resolves within a few months, our impression is that lutein supplementation prevents chronic CSCR with faster improvement. According to the report^4^ the mean height of SRD was significantly reduced by 28.6% in the oral lutein supplementation group compared with 3.3% in the placebo group. The study also revealed 30% spontaneous remission in patients with chronic CSCR. In comparison, the SRD disappearance rate with conservative treatment in our study was 63.5%, indicating the effectiveness of lutein supplementation and smoking cessation. In addition, 48.0% of chronic CSCR patients who are nonsmokers improve with lutein supplementation, which may be a greater improvement rate than simply waiting for spontaneous resolution with follow-up.

The key findings of this study indicate that chronic CSCR patients who are smokers have a more reversible SRF accumulation state and are more likely to improve with smoking cessation than CSCR patients who are nonsmokers. The effectiveness of smoking cessation as a treatment for CSCR has not been previously reported, but this is the first report showing that smoking cessation or smoking reduction can improve CSCR. Although the height of the SRD at the time of the initial visit was not correlated with visual prognosis, there was a tendency for a longer interval since the onset of SRD to be associated with poorer final visual acuity. Among CSCR patients who were currently nonsmokers, the CSCR eyes of former smokers were more likely to require PC treatment than were the CSCR eyes of patients who had never smoked (71.4% vs. 22.2%). However, the difference was not significant, probably because of the small number of cases (*p* = 0.058, Fisher’s exact test). Previous studies have shown that the CCT of CSCR patients who were current smokers was greater than that of CSCR patients who were nonsmokers^23^. However, statistical analysis revealed that the difference was not significant in our study (*p* = 0.200, Mann‒Whitney U test). The correlation between the Brinkman Index score and the risk of developing CSCR was also not significant. Many smokers do not develop CSCR, suggesting that individual tolerance to smoking or causes that have a synergistic or additive effect with smoking may be involved. In addition to CSCR, negative effects of smoking on the eye are known to include glaucoma progression^24^cataract^25^age-related macular degeneration^25^and nonarteritic anterior ischemic optic neuropathy (NA-AION)^26^. Higher BI is expected to increase the risk of these, but there are individual differences. Since many smokers do not develop CSCR, it is possible that individual tolerance to smoking or other causes of synergistic or additive effects between smoking and other risk factors that could not be excluded in this study may be involved.

There are limitations in this study, including the small number of cases and its retrospective nature. The effects of smoking cessation and lutein supplementation on CSCR could not be evaluated separately because it was not possible to establish controls in this study. In other words, it would have been more straightforward to compare the SRF resolution rates of the smoking cessation and smoking groups within the smoking cohort, rather than comparing nonsmokers to smokers, but we could not see this. The reason is that we were not able to create a group that continued to smoke or did not take lutein because we had the help of not only the patient, but also family members and a smoking cessation doctor to make sure they did not drop out. Also, it was unethical to follow up without any approach when lutein and smoking cessation could have been effective. Then, the incidence of CSCR is reported to be six times higher in male than in female^12^and in comparison, 80.0% of the CSCR patients in this statistic were also male. According to the Handbook of Health and Welfare Statistics 2022 (Ministry of Health, Labor and Welfare of Japan), the smoking rates of males and females in Japan are 25.4% for males and 7.7% for females. Although there were no female smokers among the CSCR patients in this study, the small size of the statistics does not suggest that females are more resistant to smoking. Moreover, the time of the first visit to our hospital differed from case to case, which may have resulted in differences in the treatment outcomes and speed of improvement in the CSCR. However, further studies are needed to determine the exact pathogenesis of CSCR.

## Conclusion

This study is the first to demonstrate that reducing the risk factor for smoking is a useful initial treatment for CSCR patients who smoke. Some cases of chronic CSCR are reversibly improved by smoking cessation. Lutein supplementation may also increase the rate of improvement over simply waiting for spontaneous remission. We recommend that clinicians treat patients with CSCR with lutein supplementation and smoking cessation as initial therapy.

## Electronic supplementary material

Below is the link to the electronic supplementary material.

Supplementary Material 1

## Data Availability

Data is provided within the manuscript or supplementary information files.
